# Effects of captopril and losartan on early wound healing in hypertensive rats

**DOI:** 10.1590/acb413126

**Published:** 2026-07-10

**Authors:** Anne Caroline Braz, Bruna Carla Hendges, Maria de Lourdes Pessole Biondo Simões, Sergio Ossamu Ioshii

**Affiliations:** 1 Universidade Federal do Paraná – Department of Surgery – Curitiba (PR) – Brazil.; 2 Universidade Federal do Paraná – Department of Pathology – Curitiba (PR) – Brazil.

**Keywords:** Wound Healing, Angiotensin-Converting Enzyme Inhibitors, Angiotensin II, Captopril, Losartan

## Abstract

**Purpose:**

To compare the effects of captopril and losartan on inflammatory response, angiogenesis, and fibroblast activity during the early phase of wound healing in hypertensive rats.

**Methods:**

Twenty-seven paraffin-embedded skin wound samples from hypertensive rats were analyzed: control group (n = 8), losartan-treated group (n = 9), and captopril-treated group (n = 10). Histological and immunohistochemical analyses were performed on postoperative day 4. Hematoxylin-eosin staining was used to evaluate general wound structure, and immunohistochemistry with anti-leukocyte common antigen, anti-CD34, and anti-smooth muscle actin was performed to assess inflammatory cells, angiogenesis, and fibroblast activity, respectively.

**Results:**

No significant differences were observed in angiogenesis (*p* > 0.05) or fibroblast number (*p* > 0.05) between groups. However, the inflammatory reaction was significantly higher in captopril-treated (*p* = 0.0459) and losartan-treated (*p* = 0.0452) groups compared with the control group, with no significant difference between the two treated groups.

**Conclusion:**

Neither captopril nor losartan influenced angiogenesis or fibroblast proliferation in the early healing phase. However, both drugs increased inflammatory cell infiltration.

## Introduction

The skin is the largest organ of the human body, playing essential roles in protection, thermoregulation, sensation, and vitamin D synthesis. Any trauma, whether accidental or surgical, disrupts these functions and triggers a complex healing response. Wound healing is a dynamic and complex process involving overlapping phases that restore the integrity of the tissue^1–4^.

The renin–angiotensin system (RAS), a central regulator of blood pressure, is also expressed in skin tissue and plays an important role in inflammation, fibrosis, and tissue remodeling^5,6^. Approximately 30% of the adult Brazilian population is hypertensive, and most patients are treated with antihypertensive drugs, including angiotensin-converting enzyme inhibitors (ACEIs) and angiotensin receptor blockers (ARBs)^7^.

Previous studies have shown conflicting results regarding the impact of ACEIs and ARBs on wound healing^8,9^. Some data suggest that these drugs may modulate collagen deposition, angiogenesis, and inflammation, thereby influencing healing quality^5,6,8,9^. This study aimed to compare the effects of captopril (ACEI) and losartan (ARB) on inflammatory activity, angiogenesis, and fibroblast proliferation during the early phase of wound healing in hypertensive rats.

## Methods

### Ethical approval

This study used paraffin-embedded abdominal skin wound samples previously obtained from hypertensive adult Wistar rats (*Rattus norvegicus*). All procedures followed the ethical principles for animal experimentation established by the Brazilian College of Animal Experimentation and were approved by the Animal Ethics Committee of the Universidade Federal do Paraná, protocol number 23075.027491/2023-62. The study design complied with the ARRIVE guidelines, and no human participants were involved.

### Experimental design and sample

This study consisted of a secondary histological and immunohistochemical analysis of archived paraffin-embedded skin wound samples obtained from previous experimental studies that investigated wound healing in hypertensive rats treated with antihypertensive drugs^8,9^. The experimental procedures, including hypertension induction, surgical wound creation, and animal care, were performed in those original studies and are described in detail in the corresponding publications^8,9^. The present study used the archived tissue blocks generated in those experiments for additional histological and immunohistochemical evaluation.

A total of 27 paraffin blocks containing abdominal skin wound samples were analyzed. The samples were obtained from adult male Wistar rats weighing approximately 250–300 g. Animals were housed in standard laboratory cages under controlled environmental conditions (temperature 21 ± 2°C, 12-hour light/dark cycle) with free access to food and water. Systemic hypertension was induced through the Goldblatt 2-kidney, 1-clip model. The animals were randomly allocated into three groups (n = 8 for control; n = 9 for losartan and n = 10 for captopril):

- Control group: hypertensive rats without pharmacological treatment;
- Captopril group: hypertensive rats treated with captopril 7.5 mg/kg/day;
- Losartan group: hypertensive rats treated with losartan 10 mg/kg/day.

The drugs were administered once daily via orogastric gavage for 15 consecutive days. After this treatment period, a standardized abdominal skin incision measuring approximately 4 cm was performed under anesthesia with ketamine (50 mg/kg) and xylazine (20 mg/kg) administered intraperitoneally. The incision was closed using simple interrupted sutures with 4-0 monofilament nylon.

In the original experiments^8,9^, animals were euthanized on postoperative days 4, 7, and 14. In the present study, only the specimens corresponding to postoperative day 4 were used for histological and immunohistochemical analyses.

### Histological and immunohistochemical analysis

Sections of 5 μm thickness were obtained from each paraffin block. Sections were deparaffinized in xylene and rehydrated through graded ethanol solutions. Antigen retrieval was performed using citrate buffer (pH 6.0) in a heated water bath. Endogenous peroxidase activity was blocked with hydrogen peroxide prior to incubation with primary antibodies. Slides were incubated with primary antibodies overnight at 4°C.

These initial sections were used to guide the selection of areas to be processed with immunohistochemical markers. A tissue array technique was employed, in which fragments of each sample were allocated on the same slide in an ordered and numbered fashion, allowing for standardized processing and analysis. New sections were then prepared and mounted on slides for staining.

Hematoxylin-eosin (HE) staining was performed to assess general wound morphology and inflammatory response. Immunohistochemistry was carried out using primary antibodies against leukocyte common antigen (LCA), for inflammatory cells, CD34 (endothelial cells and angiogenesis), and smooth muscle actin (SMA), for fibroblasts/myofibroblasts. All antibodies were applied according to the manufacturer’s instructions, with appropriate positive and negative controls.

### Quantitative immunohistochemical analysis

For each sample, 10 histological sections were analyzed. Three non-overlapping microscopic fields were evaluated in each section within the central wound area, avoiding wound edges and necrotic regions. Positive staining was identified by brown cytoplasmic labeling (LCA and α-SMA) or endothelial cell membrane staining (CD34). Counts were performed manually by a blinded examiner. The mean value per animal was calculated from the analyzed fields, and group means were obtained from these individual averages. Neovascularization analysis was performed on slides stained with anti-CD34.

Three random fields per section were selected, each with an area of 131,307.264 μm^2^, at 10× magnification. Digital images were acquired using the Axio Scan.Z1 Digital Slide Scanner (Zeiss, Germany). The selected fields were evaluated using Zeiss ZenLite software (Zeiss, Germany), and the number of CD34-positive vessels was counted. The mean vessel density per animal was obtained from the analyzed microscopic fields. Image acquisition parameters were kept constant for all groups.

The same procedure was applied to inflammatory cells (anti-LCA) and fibroblasts (anti-SMA). For each animal, the mean cell count was calculated from the analyzed microscopic fields, and the group mean was then determined from these individual values.

### Statistical analysis

All data are presented as mean ± standard deviation (SD). The normality of data distribution was assessed using the D’Agostino and Pearson’s and Kolmogorov-Smirnov’s tests. Intergroup comparisons were performed using unpaired Student’s t-test. A significance level of *p* ≤ 0.05 was adopted.

The statistical analysis followed established procedures (GraphPad Prism v8.0.2, GraphPad Software Inc., United States of America). The main author assumes full responsibility for the accuracy and reproducibility of all data and statistical calculations.

## Results

### Inflammatory response

**Figure 1:**
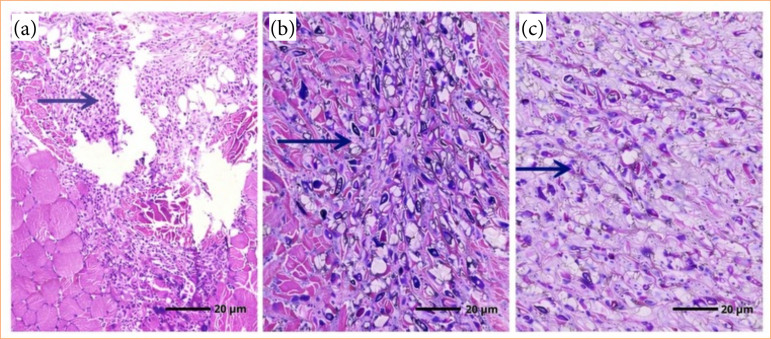
Representative histological images of inflammatory infiltrate. (a) Control group, (b) captopril-treated group, and (c) losartan-treated group. Arrows indicate areas of inflammatory cell infiltration in the wound region. Hematoxylin and eosin staining. Scale bar = 20 μm. Original magnification ×400.

Source: Elaborated by the authors.

The inflammatory response was predominantly acute–chronic with moderate intensity in all experimental groups (Fig. 1). The control group presented a mean of 12.50 ± 4.44 inflammatory cells per microscopic field, whereas the losartan and captopril groups showed means of 18.41 ± 6.39 and 17.40 ± 5.02, respectively (Table 1).

**Table 1:** Quantification of inflammatory cells assessed by anti-leukocyte common antigen immunohistochemistry. Values represent the mean number of inflammatory cells per microscopic field in the control, captopril-treated, and losartan-treated groups. Data are expressed as mean ± standard deviation.

Animal	Control	Losartan	Captopril
1	17.67	28.00	19.33
2	8.33	16.67	17.67
3	8.67	9.00	22.67
4	8.33	21.33	25.67
5	17.67	9.67	11.67
6	17.67	17.33	12.67
7	9.67	25.67	9.67
8	12.00	17.67	15.67
9	–	20.33	19.67
10	–	–	19.33
Mean ± standard deviation	12.50 ± 4.44	18.41 ± 6.39	17.40 ± 5.02

Source: Elaborated by the authors.

**Figure 2:**
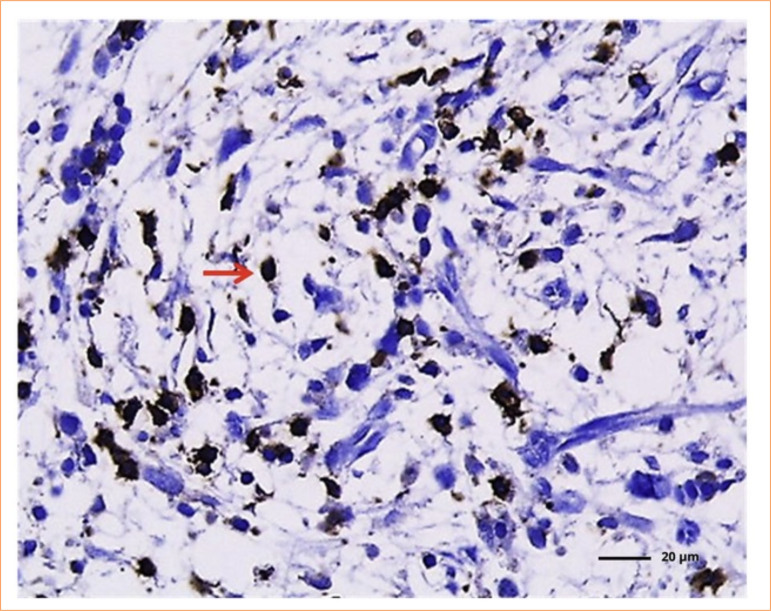
Representative immunohistochemical staining for leukocyte common antigen (LCA) showing inflammatory cell infiltration in the wound area. Arrows indicate LCA-positive inflammatory cells. Scale bar = 20 μm. Original magnification ×400.

Source: Elaborated by the authors.

Representative histological (HE) and immunohistochemical (LCA) images are presented in Figs. 1 and 2, respectively.

**Figure 3:**
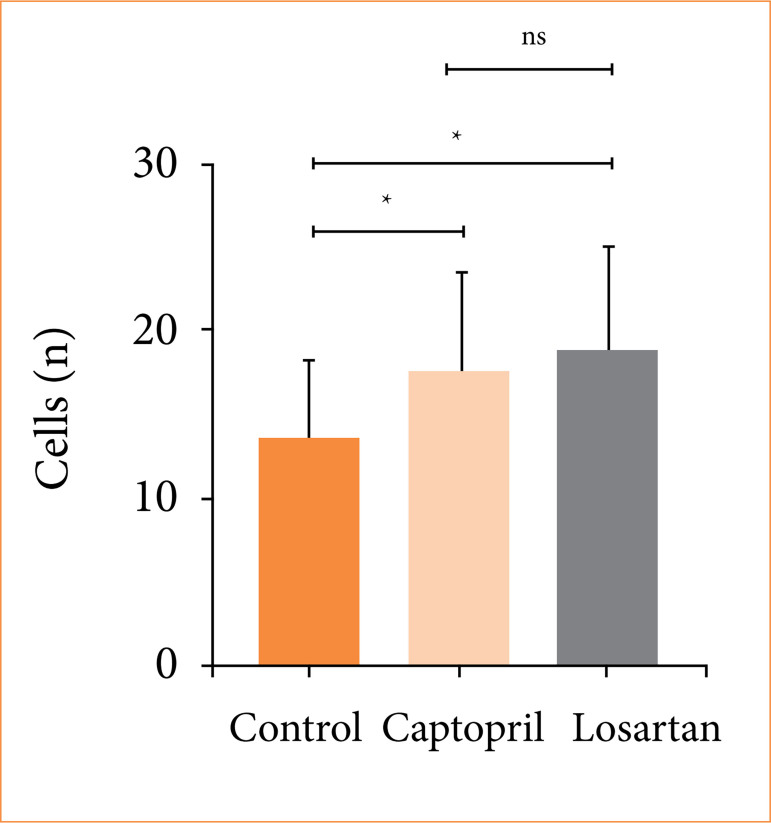
Quantitative analysis of inflammatory cells identified by anti-leukocyte common antigen immunohistochemistry. Bars represent the mean number of inflammatory cells per microscopic field in the control, captopril-treated, and losartan-treated groups. Error bars indicate standard deviation.

Quantitative analysis demonstrated a significantly higher number of inflammatory cells in both the losartan- and captopril-treated groups compared with the control group (*p* = 0.0452 and *p* = 0.0459, respectively). No statistically significant difference was observed between the treated groups (*p* = 0.7060) (Fig. 3).

*p < 0.05 compared with the control group; ns: not significant. Source: Elaborated by the authors.

### Angiogenesis

No statistically significant differences were observed in angiogenesis among the experimental groups. The mean number of CD34-positive vessels per microscopic field was 17.25 ± 3.77 in the control group, 17.37 ± 4.36 in the losartan group, and 15.57 ± 5.12 in the captopril group (*p* > 0.05) (Table 2).

**Table 2:** Quantification of angiogenesis assessed by CD34 immunohistochemistry. Values represent the mean number of CD34-positive blood vessels per microscopic field in the control, captopril-treated, and losartan-treated groups. Data are expressed as mean ± standard deviation.

Animal	Control	Losartan	Captopril
1	22.00	17.33	16.33
2	11.67	24.00	13.67
3	15.67	12.00	18.33
4	17.33	18.00	24.00
5	19.67	10.00	12.67
6	17.00	19.67	10.00
7	21.67	17.33	10.67
8	13.00	21.67	24.00
9	–	16.33	14.67
10	–	–	11.33
Mean ± standard deviation	17.25 ± 3.77	17.37 ± 4.36	15.57 ± 5.12

Source: Elaborated by the authors.

**Figure 4:**
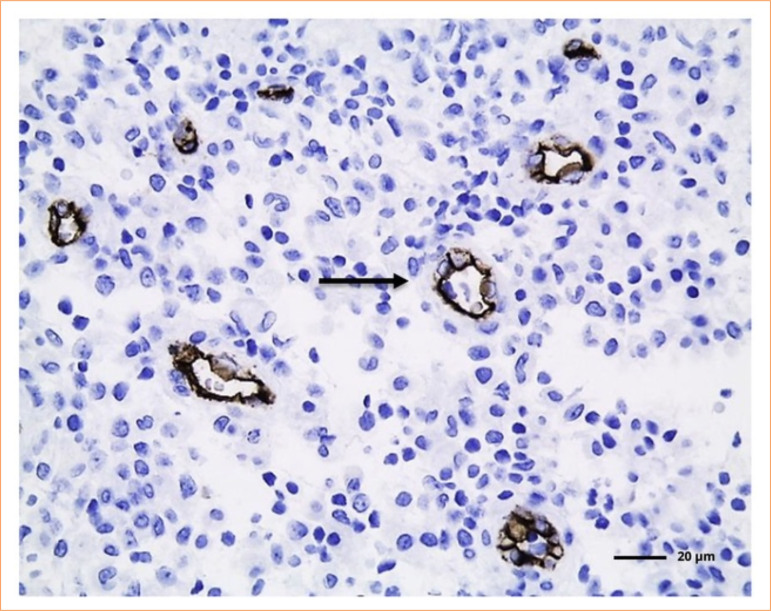
Representative immunohistochemical staining for CD34 illustrating blood vessels in the wound region. Arrows indicate CD34-positive endothelial cells forming vascular structures. Scale bar = 20 μm. Original magnification ×400.

Source: Elaborated by the authors.

**Figure 5:**
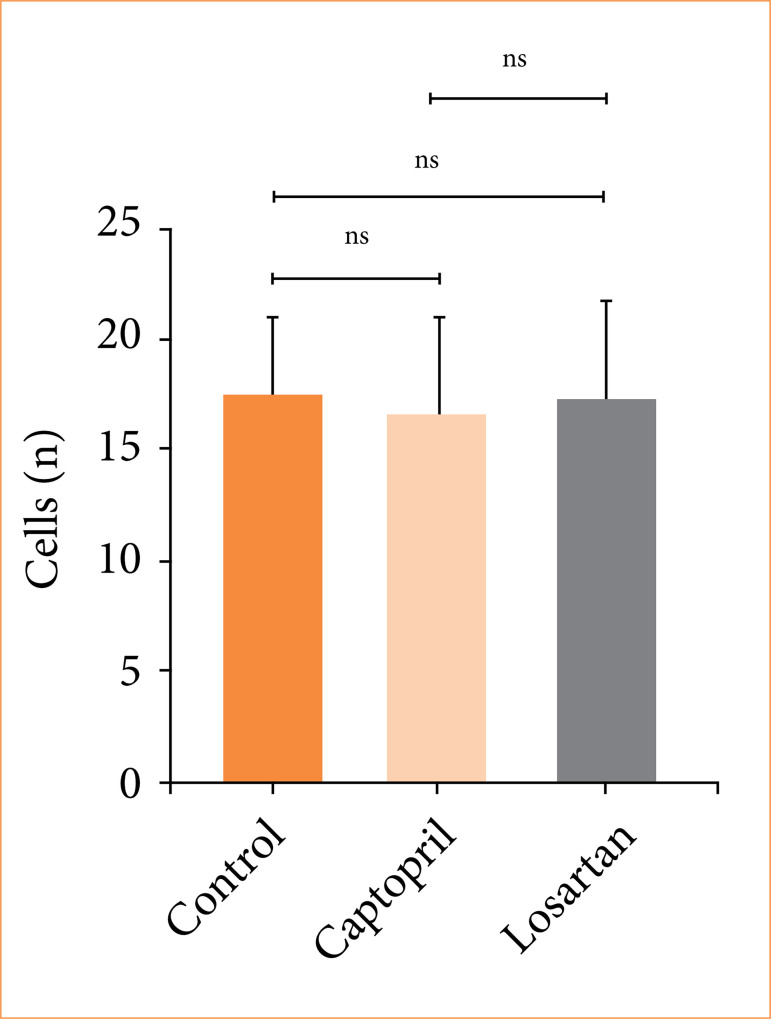
Quantitative analysis of angiogenesis assessed by CD34 immunohistochemistry. Bars represent the mean number of CD34-positive vessels per microscopic field in the control, captopril-treated, and losartan-treated groups. Error bars indicate standard deviation. No statistically significant differences were observed between groups (unpaired Student’s t-test).

Source: Elaborated by the authors.

Representative photomicrographs and quantitative analysis of angiogenesis are presented in Figs. 4 and 5.

### Fibroblast activity

Similarly, no statistically significant differences were found in myofibroblast density among the groups. The control group showed a mean of 29.71 ± 9.04 α-SMA–positive myofibroblasts per microscopic field, while the losartan and captopril groups presented means of 26.89 ± 11.06 and 26.53 ± 11.57, respectively (*p* > 0.05) (Table 3).

**Table 3:** Quantification of myofibroblasts assessed by α-smooth muscle actin (α-SMA) immunohistochemistry. Values represent the mean number of α-SMA–positive myofibroblasts per microscopic field in the control, captopril-treated, and losartan-treated groups. Data are expressed as mean ± standard deviation.

Animal	Control	Losartan	Captopril
1	44.00	46.67	43.00
2	18.00	31.00	24.00
3	25.00	22.00	31.67
4	29.33	41.67	47.33
5	32.67	22.00	21.33
6	39.67	17.67	17.67
7	29.33	15.67	12.33
8	19.67	17.33	29.33
9	–	28.00	24.33
10	–	–	14.33
Mean ± standard deviation	29.71 ± 9.04	26.89 ± 11.06	26.53 ± 11.57

Source: Elaborated by the authors.

**Figure 6:**
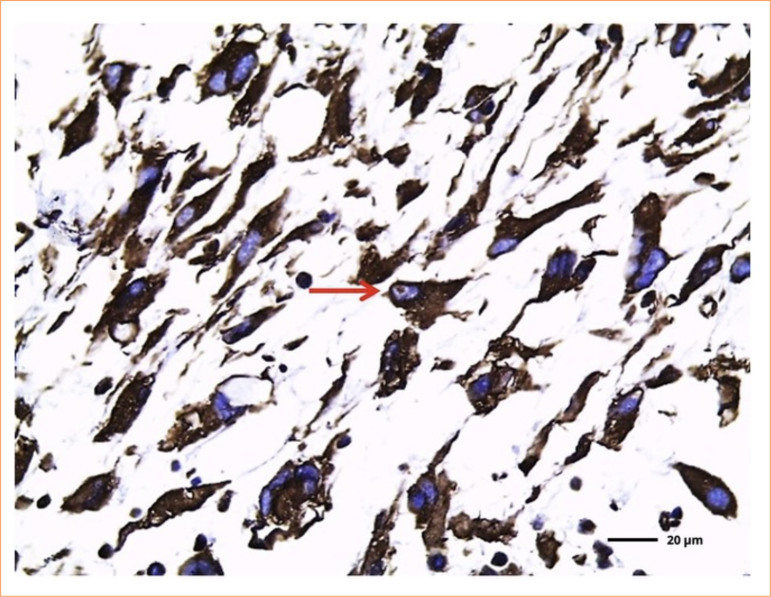
Representative immunohistochemical staining for α-smooth muscle actin (α-SMA) demonstrating myofibroblasts in the wound region. Arrows indicate α-SMA–positive myofibroblasts. Scale bar = 20 μm. Original magnification ×400.

Source: Elaborated by the authors.

**Figure 7:**
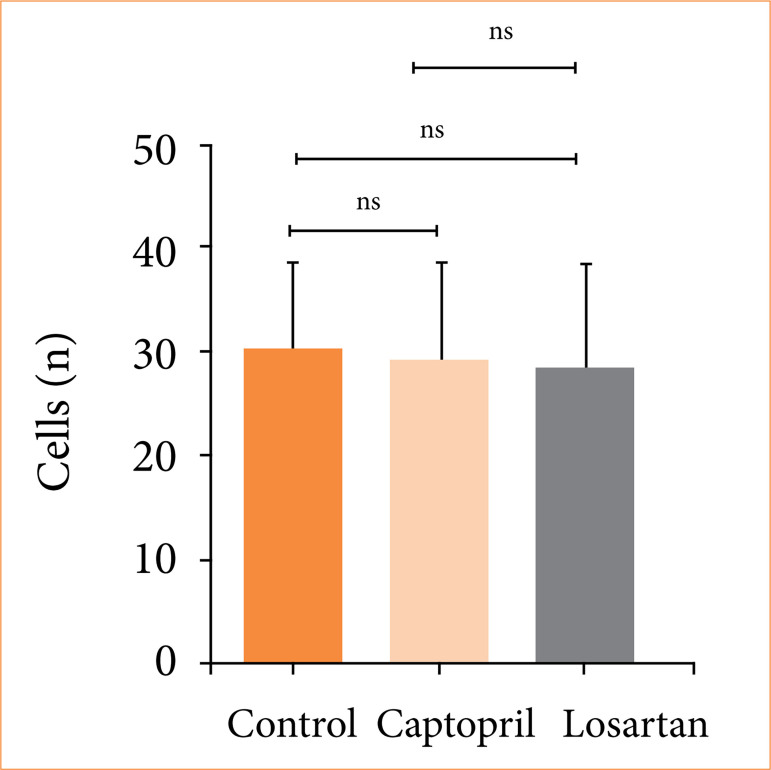
Quantitative analysis of myofibroblasts identified by α-smooth muscle actin (α-SMA) immunohistochemistry. Bars represent the mean number of α-SMA–positive myofibroblasts per microscopic field in the control, captopril-treated, and losartan-treated groups.

Representative photomicrographs and quantitative analysis of α-SMA–positive myofibroblasts are shown in Figs. 6 and 7.

*Error bars: standard deviation; ns: not significant (unpaired Student’s t-test). Source: Elaborated by the authors.

## Discussion

This study demonstrated that treatment with captopril or losartan increased inflammatory cell infiltration on postoperative day 4 in hypertensive rats, without significantly affecting angiogenesis or fibroblast proliferation at this early stage of wound healing. These findings support the concept that pharmacological modulation of the RAS may influence early inflammatory events during tissue repair.

Wound healing is a dynamic and tightly regulated process involving overlapping inflammatory, proliferative, and remodeling phases^1–3^. The inflammatory phase predominates during the first three to five days after injury and is essential for debris removal, microbial defense, and the release of cytokines and growth factors that orchestrate subsequent angiogenesis and extracellular matrix deposition^2–4^. However, excessive or dysregulated inflammation may delay the transition to the proliferative phase and compromise tissue remodeling^4^.

The skin possesses an intrinsic and functional RAS capable of locally producing angiotensin II and expressing both AT1 and AT2 receptors^5,10^. Angiotensin II is known to modulate inflammatory signaling, oxidative stress, fibroblast activation, and extracellular matrix remodeling^5,6^. ACE inhibitors reduce angiotensin II formation, whereas ARBs selectively block AT1 receptors, potentially allowing unopposed AT2 receptor activation. Experimental evidence suggests that AT2 receptor stimulation may exert anti-inflammatory effects through inhibition of NF-κB pathways^11^. Nevertheless, the net biological effect of RAS blockade in hypertensive conditions may differ from that observed in normotensive models.

Experimental studies conducted in hypertensive rat models have previously investigated the influence of antihypertensive drugs on cutaneous wound healing. Biondo-Simões et al.^8^ demonstrated that systemic arterial hypertension may alter the inflammatory and reparative phases of healing. In a subsequent experimental study evaluating the effects of losartan, the authors observed that pharmacological blockade of the angiotensin II AT1 receptor did not significantly impair the healing process but could modify inflammatory and vascular responses^9^. These findings are partially consistent with the present results, in which pharmacological modulation of the RAS did not significantly affect angiogenesis or fibroblast proliferation during the initial stage of tissue repair but was associated with increased inflammatory cell infiltration.

Other experimental studies have reported heterogeneous effects of modulation of the renin–angiotensin pathway on wound healing. While some investigations suggest that ACE inhibitors and angiotensin receptor blockers may reduce fibrosis and collagen deposition during later phases of tissue repair, others have not demonstrated significant changes in early inflammatory or angiogenic responses. These divergent findings may reflect differences in experimental models, evaluation time points, and the presence of systemic hypertension, all of which influence local RAS activity and inflammatory signaling^5,6^.

In the present study, both captopril and losartan increased inflammatory cell counts. One possible explanation is that, in the context of renovascular hypertension induced by the Goldblatt 2-kidney, 1-clip model, systemic RAS activation is markedly elevated. Pharmacological blockade may disrupt compensatory local signaling mechanisms, leading to altered leukocyte recruitment and inflammatory modulation. Previous experimental studies have reported variable effects of ACE inhibitors and ARBs on skin healing in hypertensive rats^8,9^, suggesting that the interaction between hypertension and RAS modulation is complex and model dependent.

Despite the increased inflammatory response, no significant differences were observed in angiogenesis. Angiogenesis during wound healing is primarily driven by hypoxia-inducible factors and vascular endothelial growth factor with neovascularization typically peaking during the proliferative phase3. Although angiotensin II has been implicated in endothelial proliferation and vascular remodeling^5,6^, early time-point evaluation (day 4) may not adequately capture potential differences in neovascularization kinetics.

Similarly, no significant differences were found in α-SMA–positive myofibroblast density among the groups. Myofibroblast differentiation is closely related to transforming growth factor (TGF)-β signaling and mechanical tension within the wound environment^3^. ACE inhibitors and ARBs have been shown to modulate fibrotic pathways and reduce collagen deposition in different tissues^6,12^. However, these antifibrotic effects are typically more evident during later stages of healing, particularly during extracellular matrix remodeling rather than the early inflammatory phase assessed in this study.

Recent evidence indicates that pharmacological modulation of the RAS may influence scar formation and fibrosis through canonical and noncanonical TGF-β pathways^12^. Moreover, losartan has been investigated as a therapeutic strategy for hypertrophic scars and keloids^13,14^. Nonetheless, other studies have demonstrated that early administration of captopril does not significantly improve acute wound healing outcomes^15^. These heterogeneous findings highlight that the effects of RAS-targeting drugs depend on timing, dosage, tissue type, and the presence of systemic hypertension.

From a translational perspective, these results may have relevance considering the high prevalence of hypertension and widespread use of ACE inhibitors and ARBs in surgical patients^7^. Although these medications are generally maintained perioperatively, their potential influence on early inflammatory responses at surgical wound sites warrants further investigation. An increased inflammatory infiltrate does not necessarily indicate impaired healing; in some contexts, robust early inflammation may facilitate proper progression to the proliferative phase. However, excessive or prolonged inflammation could theoretically alter scar quality or healing kinetics.

This study has limitations. First, only a single early time point (postoperative day 4) was analyzed, preventing evaluation of collagen deposition, tensile strength, and long-term remodeling. Second, quantitative assessment relied on immunohistochemical cell counting rather than molecular analysis of inflammatory mediators or profibrotic signaling pathways. Third, although the Goldblatt model reliably induces renovascular hypertension, it may not fully reproduce the multifactorial nature of essential hypertension in humans.

Future studies should include later time points (days 8–15 and beyond), evaluate collagen organization and biomechanical properties of the scar, and investigate molecular mediators such as TGF-β, vascular endothelial growth factor, interleukin-6, and tumor necrosis factor-α. Comparative analyses between normotensive and hypertensive animals under identical pharmacological treatment would also clarify whether the observed inflammatory increase is primarily drug-related or hypertension-dependent.

## Conclusion

Captopril and losartan increased inflammatory cell infiltration during the early phase of wound healing in hypertensive rats but did not significantly affect angiogenesis or myofibroblast density on postoperative day 4. These findings indicate that modulation of the RAS influences early inflammatory dynamics without significantly altering proliferative parameters at this stage of healing. Further studies evaluating later stages of healing are necessary to determine potential long-term effects on tissue remodeling.

## Data Availability

Data will be available from the corresponding author upon reasonable request.
